# SGFNet: Redundancy-Reduced Spectral–Spatial Fusion Network for Hyperspectral Image Classification

**DOI:** 10.3390/e27100995

**Published:** 2025-09-24

**Authors:** Boyu Wang, Chi Cao, Dexing Kong

**Affiliations:** 1 Faculty of Innovation and Engineering, Macau University of Science and Technology, Taipa 999078, Macau; 2240004506@student.must.edu.mo (B.W.); 2240004659@student.must.edu.mo (C.C.); 2 School of Mathematical Sciences, Zhejiang University, Hangzhou 310027, China

**Keywords:** hyperspectral image classification, spectral spatial fusion, redundancy-reduced, spectral aware filtering

## Abstract

Hyperspectral image classification (HSIC) involves analyzing high-dimensional data that contain substantial spectral redundancy and spatial noise, which increases the entropy and uncertainty of feature representations. Reducing such redundancy while retaining informative content in spectral–spatial interactions remains a fundamental challenge for building efficient and accurate HSIC models. Traditional deep learning methods often rely on redundant modules or lack sufficient spectral–spatial coupling, limiting their ability to fully exploit the information content of hyperspectral data. To address these challenges, we propose SGFNet, which is a spectral-guided fusion network designed from an information–theoretic perspective to reduce feature redundancy and uncertainty. First, we designed a Spectral-Aware Filtering Module (SAFM) that suppresses noisy spectral components and reduces redundant entropy, encoding the raw pixel-wise spectrum into a compact spectral representation accessible to all encoder blocks. Second, we introduced a Spectral–Spatial Adaptive Fusion (SSAF) module, which strengthens spectral–spatial interactions and enhances the discriminative information in the fused features. Finally, we developed a Spectral Guidance Gated CNN (SGGC), which is a lightweight gated convolutional module that uses spectral guidance to more effectively extract spatial representations while avoiding unnecessary sequence modeling overhead. We conducted extensive experiments on four widely used hyperspectral benchmarks and compared SGFNet with eight state-of-the-art models. The results demonstrate that SGFNet consistently achieves superior performance across multiple metrics. From an information–theoretic perspective, SGFNet implicitly balances redundancy reduction and information preservation, providing an efficient and effective solution for HSIC.

## 1. Introduction

Each pixel in a hyperspectral image (HSI) contains continuous spectral information, giving hyperspectral imaging techniques stronger object recognition capabilities than traditional RGB methods [1]. In recent years, hyperspectral imaging has been widely applied in fields such as crop identification [2], mineral exploration [3], and environmental monitoring [4]. Among the various tasks based on HSI processing, hyperspectral image classification (HSIC) is one of the core research directions [5,6,7]. HSIC mainly predicts the category labels of different objects. Due to its significant practical application value, HSIC has always been a hot research topic in the field of remote sensing [8,9,10].

Previously, supervised learning-based machine learning methods were applied to HSIC, such as the K Nearest-Neighbor algorithm (KNN) [11] and the support vector machine (SVM) [12]. However, these machine learning algorithms rely on manual feature extraction, making it difficult to automatically learn complex spectral and spatial features and weakening the ability to model spatial information. On the other hand, with the emergence of deep learning technology in recent years, the above problems have been effectively solved. Deep learning can automatically learn complex spectral features and spatial structures and mine deeper nonlinear relationships, thus achieving better results in HSIC, and it has therefore become the focus of many scholars in modern HSIC [13,14,15].

In the research process of applying deep learning to HSIC, many convolutional neural network (CNN) models have been used due to their excellent ability for local feature extraction [16]. A CNN can effectively capture the local spatial correlation in an image through a convolution operation. With the help of multi-layer convolution and pooling operations, it can gradually extract the abstract features from the low to the high level, showing outstanding advantages in the spatial feature processing of HSIs [17,18,19]. At an early stage, Chen et al. [20] used a regularized deep feature extraction (FE) method based on a CNN, which utilized multiple convolutional and pooling layers to extract nonlinear, discriminative, and invariant deep features of HSIs, effectively extracting spectral–spatial features of HSIs and avoiding the problem of overfitting. To solve the problems of excessive computational complexity in 3D-CNNs and the inability to fully utilize spectral information in 2D-CNNs, Roy et al. [21] proposed the Hybrid Spectral Convolutional Neural Network (HybridSN), which makes use of the hybrid structures of 3D-CNNs and 2D-CNNs and balances the spectral–spatial feature extraction and computational efficiency by extracting the spectral–spatial features first and then refining the spatial features. Chen et al. [22] proposed for the first time an auto-convolutional neural network (Auto-CNN) for HSIC, which utilizes one-dimensional auto-convolutional neural networks and three-dimensional auto-convolutional neural networks as spectral and spectral spatial classifiers, respectively, to solve the problem that manually designed deep learning architectures may not be able to adapt to a specific dataset.

Although CNNs have many advantages in HSIC, they still have limitations. Because the sensory field in the convolution limits the CNN, capturing long-distance dependencies and global context information in an HSI is difficult, and the processing of spectral features is relatively singular [23]. Therefore, many scholars have focused on the Transformer model to overcome the shortcomings of CNN in HSIC. Hong et al. [24] introduced the Transformer into HSIC for the first time and proposed a novel network called SpectralFormer. It uses neighboring bands in the HSI to learn spectral local sequence information. It generates group spectral embeddings, which are connected by cross-layer jumps to transfer class memory components, thus better mining spectral feature sequence attributes to reduce the loss of key information. In order to solve the problem of most deep learning-based HSIC methods destroying spectral information when extracting spatial features or only being able to extract sequential spectral features in short-range contexts, Ayas et al. [25] proposed a network architecture called SpectralSWIN, which makes use of the proposed Swin-Spectral Module to extract spectral–spatial features by combining the sliding-window self-attention and grouped convolution of spectral dimensions to achieve the hierarchical extraction of spectral–spatial features. Simultaneously, in order to address the issues of excessive dimensionality, spectral information redundancy, and the difficulty of appropriately combining spatial and spectral information in HSI, including the failure of existing methods to utilize first-order derivatives and frequency domain information fully, Fu et al. [26] proposed a Differential-Frequency Attention-based Band Selection Transformer (DFAST) architecture by using the DFASEmbeddings module containing a multi-branching structure, a 3D convolution, the spectral–spatial attention mechanism, and the cascade Transformer encoder. A Differential-Frequency Attention-based Band Selection Transformer (DFAST) architecture is proposed.

Although Transformers demonstrate remarkable advantages in capturing global dependencies for HSI, they still suffer from high computational overhead, particularly when handling long sequences, since the self-attention mechanism scales quadratically with sequence length [27]. Recently, Mamba [28], an emerging sequence modeling paradigm, has offered a new direction for HSIC research. Building on this, Sun et al. [29] proposed the Hyperspectral Spatial Mamba (HyperSMamba) model, which integrates the Spatial Mamba (MS-Mamba) encoder with an Adaptive Fusion Attention Module (AFAttention). This design not only alleviates the quadratic complexity bottleneck of Transformer-based self-attention but also mitigates the excessive computational burden in prior Mamba-based approaches, which is caused by selective scanning strategies.

Although the Mamba model has improved computational complexity compared to the Transformer, we can see from the above models that the Mamba architecture is primarily based on the state space model (SSM) [30,31,32]. However, Yu et al. [33] found that SSM contains a significant amount of redundancy in image classification tasks, which negatively impacts classification accuracy. Therefore, they proposed the MambaOut model, which removes SSM from the model, significantly reducing the computational power required while improving performance. Additionally, we found that many deep learning models used for HSIC address the issue of excessive computational requirements by performing dimension reduction via principal component analysis (PCA) [34]. However, this also poses the problem of partial spectral feature loss [35].

Therefore, to address the challenges in HSIC, we explored whether key spectral information could be extracted through independent modules after PCA dimensionality reduction to compensate for spectral information loss. Could a structure similar to Mamba be designed to achieve an efficient fusion of spectral and spatial features while enhancing computational efficiency? Thus, we propose the Spectral-Guided Fusion Network (SGFNet). Its core component is the Spectral-Guided Gated Convolutional Network (SGGC), which draws inspiration from the essence of the Mamba model while eliminating its redundant State Space Mechanism (SSM). To precisely capture the complex interplay between spectral and spatial information, we first process guiding spectral features through a Spectral-Aware Filtering Module (SAFM). This module encodes raw spectral sequences into a globally shared representation while amplifying critical spectral information. These spectral features are then fed into a redesigned Mamba architecture to compensate for key spectral information lost during PCA processing. To effectively fuse one-dimensional spectral features with two-dimensional spatial features, we introduce the Spectral–Spatial Adaptive Fusion (SSAF) module, enabling the efficient integration of spectral representations with global spatial representations. The fused features are applied to the gating mechanism of the SGGC, enabling spectral features to more effectively guide spatial feature extraction. Through rigorous comparative experiments, the proposed SGFNet demonstrates outstanding classification performance. In summary, our contributions include the following:

1. We propose SGFNet, which is an innovative spectral-guided MambaOut network for hyperspectral image classification (HSIC). From an information–theoretic perspective, hyperspectral data often exhibit high spectral redundancy, resulting in high entropy and making it challenging to extract discriminative features. SGFNet explores the feasibility of the MambaOut architecture in HSIC and demonstrates that the state–space mechanism (SSM) is not indispensable. By leveraging low-entropy spectral priors to guide spatial feature extraction, SGFNet enhances informative patterns while significantly reducing the number of parameters and FLOPs.
2. We design a Spectral-Aware Filtering Module (SAFM) that effectively suppresses redundant spectral responses while retaining informative spectral components. This process can reduce the entropy of raw hyperspectral data to provide reliable and high-information support for subsequent modules.
3. We propose the Spectral-Guided Gated CNN (SGGC), which is a Mamba-inspired structure without the SSM. Within SGGC, we introduce the Spectral–Spatial Adaptive Fusion (SSAF) module, which aggregates one-dimensional spectral features with two-dimensional spatial features and feeds the fused, low-entropy representations into the gating mechanism, effectively guiding spatial feature extraction with reduced uncertainty through spectral information.
4. We conducted rigorous and fair comparative experiments. The experimental results show that SGFNet achieved the highest overall accuracy (OA), average accuracy (AA), and Kappa coefficient when compared with eight state-of-the-art algorithms on four benchmark datasets. These results demonstrate the effectiveness of our entropy-aware design in improving HSIC performance.

The remainder of this paper is organized as follows. In Section 2, we introduce related work. In Section 3, we describe in detail the various components of the proposed SGFNet. In Section 4, we conduct detailed experiments and analyses. Section 5 discusses and compares the effects of different parameters from multiple perspectives. Finally, Section 6 summarizes the overall model and outlines future development directions.

## 2. Related Work

### MambaOut

**Figure 1:**
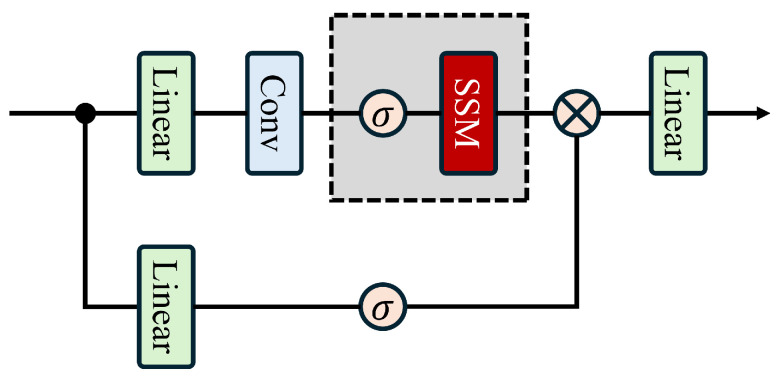
Architecture with shadow regions (Mamba), architecture without shadow regions (Gated CNN).

MambaOut is an architecture that removes the state-space model (SSM), and experiments demonstrate its superiority over the visual Mamba model in the ImageNet image classification task. Its core module is the Gated Convolutional Neural Network (Gated CNN). As shown in Figure 1, this structure illustrates the comparison between the Mamba and the Gated Convolutional Neural Network. The overall architecture incorporates multiple linear transformations, convolutional operations, and gating mechanisms. When the gray dashed region is included, it corresponds to the complete Mamba architecture; removing this component yields the Gated CNN architecture. The gating mechanism efficiently captures spatial local correlations while suppressing redundant frequency bands. Consequently, MambaOut achieves a balance between feature extraction and computational efficiency. Assuming the input is *X*, the specific process is as follows:

(1) $$X^{*}=\mathrm{Norm}\left(X\right)$$

(2) $$X_{CNN}=\left(\mathrm{TokenMixer}\left(X^{*}W_{1}\right)⊙\sigma \left(X^{*}W_{2}\right)\right)W_{3}+X$$

where Norm(·) is normalized, $X^{*}$ is the normalized intermediate variable, and $X_{CNN}$ denotes the obtained variable after the gated CNN block. $W_{1}$, $W_{2}$, and $W_{3}$ are learnable parameters obtained by linear operations.$\;\mathrm{TokenMixer}\left(\cdot \right)$ is the convolution operation.

## 3. Methods

### 3.1. Spectral-Aware Filter Module

The SAFM we designed aims to extract more recognisable spectral features from HSI and select important spectral frequency information in the frequency domain. From an information–theoretic perspective, SAFM effectively reduces redundant spectral responses, lowering the entropy of the spectral representation while preserving critical discriminative information. By suppressing high-entropy (noisy or redundant) components and retaining informative low-entropy features, SAFM provides a more compact and informative spectral encoding for subsequent modules. The process is as follows: first, we assume that the original spectral feature is $S_{in}$, and then we apply the Fast Fourier Transform (FFT) to obtain its frequency-domain representation:

(3) $$S_{in}^{f}=F\left(S_{in}\right)$$

Next, we modulate the frequency components using a quantization matrix W and transform back to the time domain:

(4) $$S_{1}=F^{-1}\left(WS_{in}^{f}\right)$$

Finally, the filtered spectral feature $S_{1}$ is passed through a multi-layer perceptron (MLP) to produce the output $S_{out}$:

(5) $$S_{out}=\sigma_{\mathrm{out}}\left(W_{L+1}^{\prime }\cdot \sigma \left(W_{L}^{\prime }\cdot \sigma \left(\ldots \sigma \left(W_{1}^{\prime }S_{1}+b_{1}\right)+b_{2}\right)\ldots +b_{L}\right)+b_{L+1}\right)$$

where $W_{k}^{\prime }$ and $b_{k}$ are the weight matrix and bias vector of the *k*-th layer, σ(·) is the hidden layer activation function, and $\sigma_{\mathrm{out}}\left(\cdot \right)$ is the output layer activation function.

From an information–theoretic perspective, the SAFM effectively reduces redundant or noisy spectral components—corresponding to high-entropy information in the frequency domain—while retaining low-entropy, informative spectral features. The quantization matrix W acts to suppress high-entropy components, and the subsequent MLP integrates the filtered spectral features into a compact and discriminative representation suitable for downstream spectral–spatial processing.

### 3.2. Spectral–Spatial Adaptive Fusion Module

**Figure 2:**
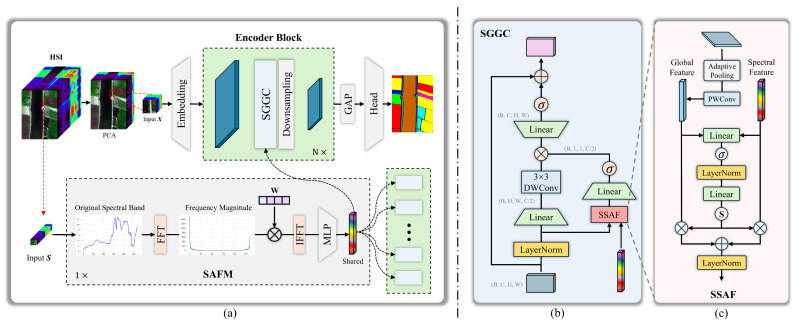
Detailed flowchart of SGFNet. (**a**): The upper part is the main branch of SGFNet, and the lower part is the Spectral-Aware Filtering Module (SAFM). (**b**): Spectral-Guided Gated CNN Module (SGGC). (**c**): Spectral–Spatial Adaptive Fusion Module (SSAF).

The precise fusion of spatial and spectral features is crucial in HSIC tasks. However, existing models for HSIC often employ direct addition or concatenation methods for feature fusion, which struggle to capture the spatial–spectral correlations between different regions accurately. Therefore, we propose a solution called the SSAF module, which can dynamically generate spectral–spatial weights and perform subsequent fusion processes to integrate two-dimensional spatial features and one-dimensional spectral features accurately; the process is illustrated in Figure 2c. Assuming that SSAF takes spatial features *x* and spectral features *s* as inputs, its output is the fusion feature *f*. The specific derivation process is as follows:

(6) $$x^{\prime }=\mathrm{PWConv}\left(\mathrm{AdaptiveAvgPool}\left(x\right)\right)$$

Subsequently, $x^{\prime }$ and *s* pass through their shared parameters simultaneously. The entire process is as follows:

(7) $$\begin{array}{cc} \; & w_{1}=\sigma \left({\mathrm{Linear}}_{h\to C}\left(\mathrm{LayerNorm}\left(\mathrm{GELU}\left({\mathrm{Linear}}_{C\to h}\left(x^{\prime }\right)\right)\right)\right)\right)\\ \; & w_{2}=\sigma \left({\mathrm{Linear}}_{h\to C}\left(\mathrm{LayerNorm}\left(\mathrm{GELU}\left({\mathrm{Linear}}_{C\to h}\left(s\right)\right)\right)\right)\right) \end{array}$$

where $h=\frac{C}{4}$ is the hidden dimension after dimensionality reduction, σ is the activation function, and lightweight weight calculation is achieved through a path from dimensionality reduction to nonlinear activation to dimensionality expansion. Here, weights w1 and w2 represent the importance of spatial features and spectral features in the current sample. Finally, the generated weights are used to perform weighted fusion on the input features to obtain the following:

(8) $$f=\;\mathrm{LayerNorm}\left(w_{1}⊙x^{\prime }+w_{2}⊙s\right)$$

where symbol ⊙ denotes element-wise multiplication.

### 3.3. Spectral Guidance Gated CNN Module

The SGGC module we designed has the structure shown in Figure 2b. SGGC consists of three parts: the main branch for spatial feature extraction, the gated branch of the module centred on SSAF, and the residual connection. Therefore, we set the input spatial features as $x\in R^{B\times C\times H\times W}$ and the input frequency domain features as $s\in R^{B\times C}$. First, the original features are retained as residual shortcuts to mitigate the gradient vanishing problem in deep networks. The specific process of this module is as follows.

(9) shortcut=x

And then to fit the dimension requirement of LayerNorm, the dimension order of spatial features is first adjusted, where the channel dimension is postponed to obtain the following:

(10) xtrans1=rearrangex,bhwc′→bhwc′∈RB×H×W×C

The channel features at each spatial location are also normalized to stabilize the fluctuations in the distribution due to intensity differences in the hyperspectral bands, and so the following is obtained:

(11) $$x_{\mathrm{norm}\;}\left[b,h,w,c\right]=\frac{x_{\mathrm{trans}1\;}\left[b,h,w,c\right]-\mu_{b,h,w}}{\sqrt{\sigma_{b,h,w}^{2}+\epsilon }}$$

where $\mu_{b,h,w}$ is the channel mean, $\sigma_{b,h,w}^{2}$ is the channel variance, and ϵ is the anti-zero constant. So, we derive the main branch, which first extends the normalized features to the hidden dimension h=expand×C(expand=0.5) through the linear layer to enhance the feature capacity, and so we obtain the following:

(12) $$c_{1}=W_{\mathrm{in}}\cdot x_{\mathrm{norm}}+b_{\mathrm{in}}\in R^{B\times H\times W\times h}$$

where $W_{\mathrm{in}\;}\in R^{h\times C}$ is is the weight matrix and $b_{\mathrm{in}}\in R^{h}$ is the bias. And then the following is obtained after DWConv:

(13) $$c_{2}=DWConv_{3\times 3}\left(c_{1}\right)$$

Finally, the dimensions are recovered to fit the subsequent gating operation, which is obtained as follows:

(14) c=rearrangec2,bhwc′→bhwc′∈RB×H×W×h

The second part is a gate-controlled branch, which aims to fuse spatial and spectral features to generate joint features. The specific steps are as follows:

(15) $$g_{\mathrm{fusion}\;}=\mathrm{SSAF}\left(x_{\mathrm{norm}},s\right)=\mathrm{LayerNorm}\left(w_{1}⊙x_{\mathrm{norm}}+w_{2}⊙s\right)\in R^{B\times C}$$

From Equation (7), $w_{1},w_{2}\in R^{B\times C}$ are the dynamic weights generated by the SSAF, which achieves adaptive balance between spatial and spectral features. And then the fusion features are projected to the hidden dimension *h*, and the spatial dimension is extended to match the spatial dimensions of the main branch features, which is obtained as follows:

(16) $$g=W_{g}\cdot g_{\mathrm{fusion}\;}+b_{g}\in R^{B\times h}$$

Finally, the obtained gating signal g is activated by GELU, and the main branch feature c is screened element by element and finally output by residual connection as follows:

(17) $$\begin{array}{cc} \; & x_{\mathrm{gate}\;}=\mathrm{GELU}\left(g\right)⊙c\in R^{B\times H\times W\times h}\\ \; & x_{\mathrm{out}\;}=\mathrm{GELU}\left(W_{\mathrm{out}\;}\cdot x_{\mathrm{gate}\;}+b_{\mathrm{out}}\right)\in R^{B\times H\times W\times C} \end{array}$$

where $W_{\mathrm{out}\;}\in R^{C\times h}$ is the dimensionality reduction matrix, and the output is obtained as follows:

(18) y=rearrangexout,bhwc′→bchw′+shortcut

### 3.4. SGFNet Overview

**Figure 3:**
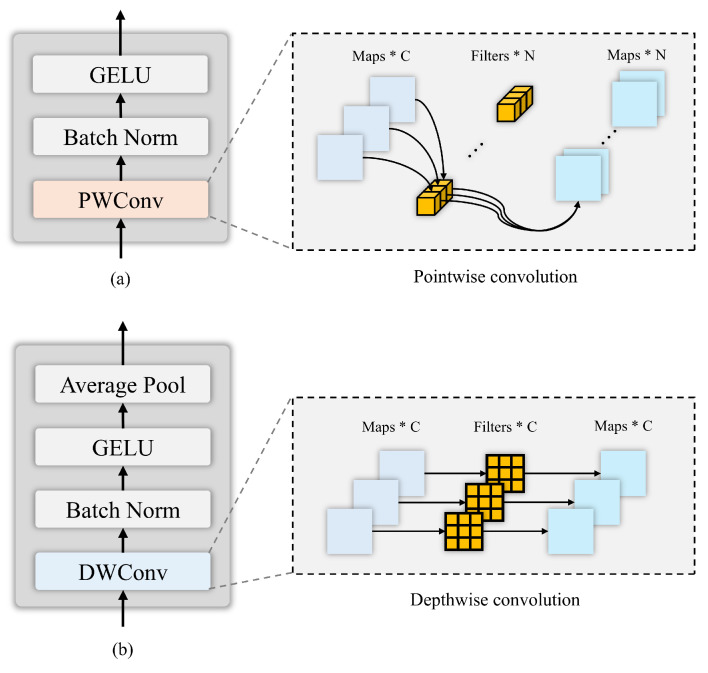
Embedding and downsampling details diagram. The asterisk ‘*’ indicates multiplicity. (**a**): Embedding layer, (**b**): downsampling layer.

The core of the SGFNet is a multi-stage spectral guided gated convolution + downsampling method that gradually extracts spatial–spectral features. The innovative shared spectral encoding ensures the injection of spectral information across stages, ultimately outputting category predictions through a classification head. The detailed process is shown in Figure 2a. We assume that the input raw spatial feature tensor is $X_{\mathrm{raw}\;}\in R^{B\times C_{\mathrm{raw}\;}\times H\times W}$, the spectral features after SAFM screening are $S_{safm}$, and the final representation is $\hat{Y}$. To reduce the dimensionality of spatial features, PCA dimension reduction is first performed on $X_{\mathrm{raw}}$. The steps are as follows:

(19) $$X=\mathrm{PCA}\left(X_{\mathrm{raw}}\right)=X_{\mathrm{raw}\;}\cdot P\in R^{B\times C_{\mathrm{in}\;}\times H\times W}$$

where $C_{\mathrm{in}\;}$ indicates the number of channels after dimensionality reduction. Moreover, $P\in R^{C_{\mathrm{raw}\;}\times C_{\mathrm{in}\;}}$ denotes the principal component analysis (PCA) projection matrix. Subsequently, we input the PCA-dimension-reduced spatial feature vector *x* into the embedding layer, whose specific details are shown in Figure 3a, to align the spatial feature dimension with the spectral feature dimension. The following results are obtained:

(20) $$X_{\mathrm{emb}}=\;\mathrm{EmbeddingLayer}\left(X\right)\in R^{B\times C_{\mathrm{emb}}\times H\times W}$$

Then, $X_{\mathrm{emb}}=x_{i}$ (i=0,1,2…·n−1) undergoes feature extraction and dimension reduction through multiple consecutive SEGC modules and downsampling layers, gradually integrating spatial and spectral features. The specific details of downsampling are shown in Figure 3b. Thus, we obtain the following:

(21) $$\begin{array}{c} x_{1}=\mathrm{DownsampleLayer}(\mathrm{SGGC}(x_{0},S_{safm})\;\\ \vdots \\ x_{n}=\mathrm{DownsampleLayer}(\mathrm{SGGC}(x_{n-1},S_{safm})\; \end{array}$$

Thus, the final spatial feature $x_{n}$ is processed by the classification head to generate category probabilities, resulting in the following:

(22) $$\begin{array}{cc} \; & x_{\mathrm{pool}\;}=\;\mathrm{AdaptiveAvgPool}2d\left(x_{n}\right)\\ \; & \hat{Y}=\mathrm{Linear}\left(\mathrm{Flatten}\left(x_{\mathrm{pool}\;}\right)\right) \end{array}$$

The specific procedural steps of the model can be obtained from Algorithm 1.
**Algorithm 1:** Pseudo-Procedure of the Proposed SGFNet
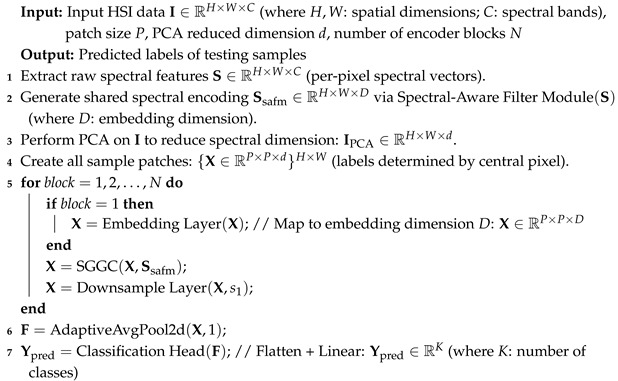


## 4. Results

This section provides a detailed introduction to the dataset used in the experiment, parameter settings, model comparison results, and visualization analysis. We chose to compare the proposed model with eight cutting-edge models commonly used in the HSIC field to evaluate its effectiveness. All tests used the same hyperparameter settings and experimental conditions as the original study to ensure fairness.

### 4.1. Data Description

To comprehensively evaluate the performance of the proposed model, we conducted comparative experiments using four well-known hyperspectral datasets: the Augsburg dataset (AU), the Houston 2013 dataset (HU2013), the Pavia University dataset (PU), and the WHU-Hi-LongKou dataset (LK). The following subsections will provide detailed information about each dataset.

#### 4.1.1. Augsburg Dataset

**Figure 4:**
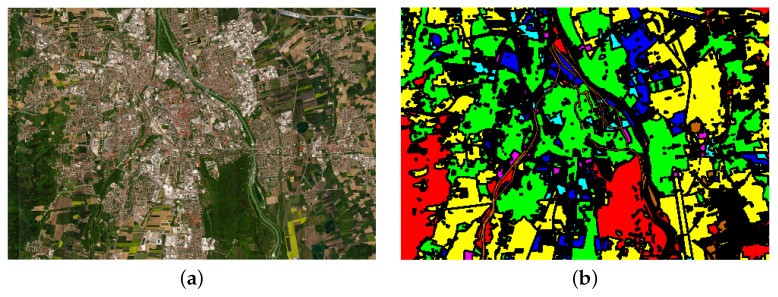
Augsburg dataset. (**a**) False color image. (**b**) Ground truth.

The Augsburg dataset collection utilized three dedicated systems: the HySpex sensor for hyperspectral imaging, the C-band synthetic aperture radar (SAR) sensor installed on the Sentinel-1 satellite, and the DLR-3K system for digital elevation model (DEM) data. The Augsburg dataset is divided into seven categories based on land cover types. The false-color image and the ground truth image of the dataset are shown in Figure 4.

#### 4.1.2. Houston2013 Dataset

**Figure 5:**
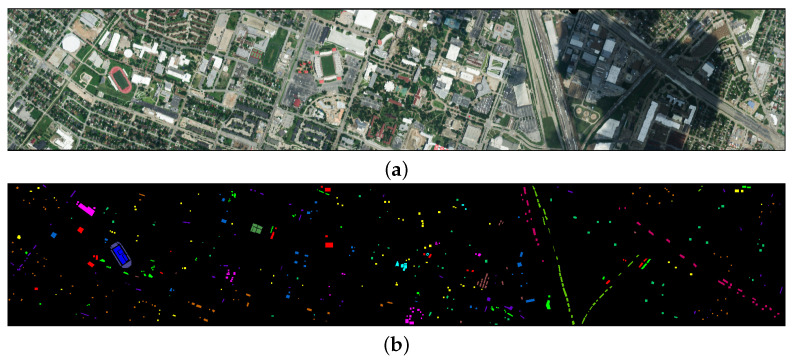
Houston2013 dataset. (**a**) False color image. (**b**) Ground truth.

The Houston 2013 dataset was acquired by the ROSIS-3 sensor near the University of Houston, Texas, USA, and has a spatial resolution of 2 meters, a spectral range of 430–860 nanometers, 144 bands, and an image size of 349 × 190 pixels, covering 15 land cover types. The false-color image and the ground truth image of the dataset are shown in Figure 5.

#### 4.1.3. Pavia University Dataset

**Figure 6:**
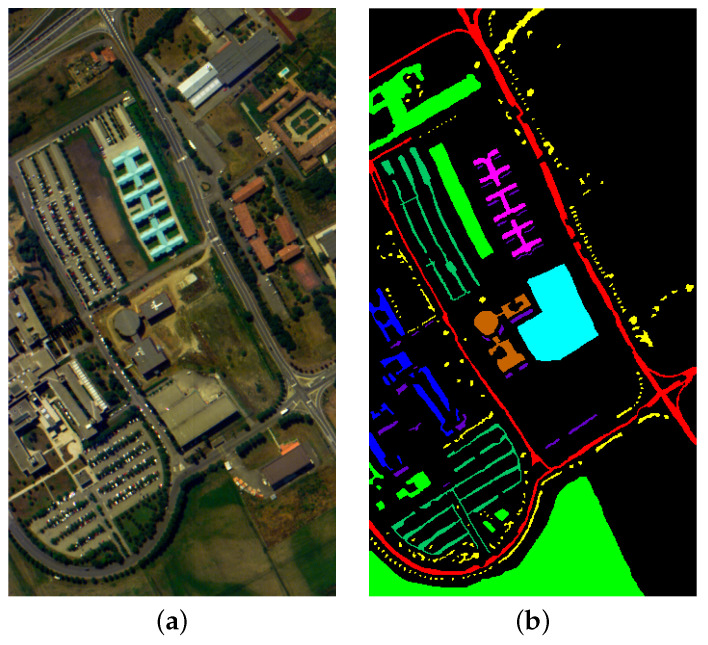
Pavia University dataset. (**a**) False color image. (**b**) Ground truth.

The Pavia University dataset was acquired using the Reflective Optical System Imaging Spectrometer (ROSIS-3) sensor in Pavia, Italy. It features images with a 610 × 340 pixel resolution across 115 spectral bands. This dataset includes 42,776 annotated samples representing nine land cover categories: asphalt, grassland, gravel, trees, metal debris, bare soil, bricks, and shadows. The false-color image and the ground truth image of the dataset are shown in Figure 6.

#### 4.1.4. WHU-Hi-LongKou Dataset

**Figure 7:**
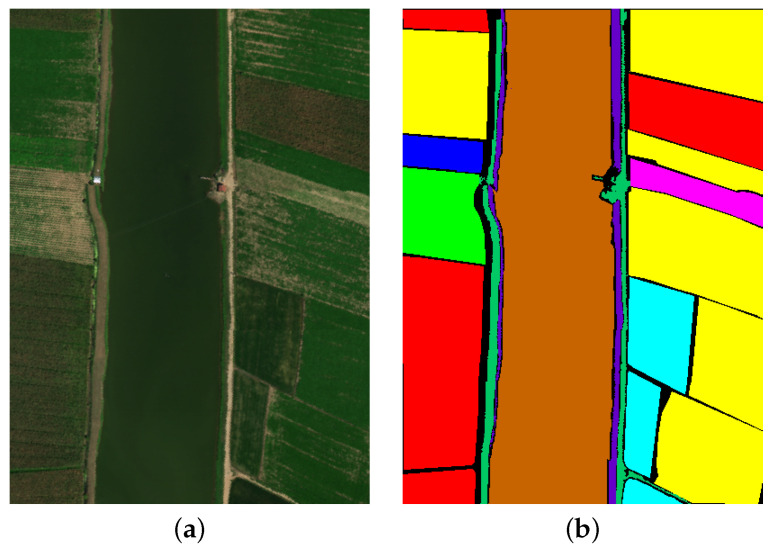
WHU-Hi-LongKou dataset. (**a**) False color image. (**b**) Ground truth.

The WHU-Hi-Longkou dataset was collected in Wuhan, Hubei Province, using Headwall Nano-Hyperspec imaging sensors. The study area covers a typical agricultural region with nine different land cover types. The dataset contains image files with a 550 × 400 pixels resolution, comprising 270 spectral bands covering a wavelength range of 400 to 1000 nanometers. The false-color image and the ground truth image of the dataset are shown in Figure 7.

### 4.2. Experimental Settings

All algorithmic modeling experiments in the article were implemented using Python 3.12.3 and Pytorch 2.5.1, and they were trained on computers equipped with RTX 4060Ti 16 GB GPU (NVIDIA, Santa Clara, CA, USA) and Intel Core i5-13600KF CPU (Intel, Santa Clara, CA, USA).

The AU, HU 2013, PU, and LK datasets each contain 30 training samples with the remaining samples forming the test set. Table 1, Table 2, Table 3 and Table 4 present the distribution of training and test samples for each land cover class within each dataset along with the total number of samples. The hyperparameters are set as follows: the initial learning rate is 0.03, The total number of training epochs for all four datasets is 200, and the learning rate is halved every 50 epochs. To optimize the model, we select the Adam optimizer. Additionally, during the experiments, to eliminate the interference of random factors on the stability of the results, we conduct independent repeated experiments using ten completely randomly generated random seeds to validate the robustness of the model performance fully.

To more thoroughly analyze and compare the classification performance of the proposed model, six commonly used evaluation metrics were selected for the experiment: classification accuracy for each feature category, overall accuracy (OA), average accuracy (AA), Kappa coefficient (Kappa), number of parameters required, and model FLOPS. Additionally, to more intuitively compare the classification performance of each model, visualization charts of the classification results for each comparison model were generated. Finally, all experimental results are based on the average of ten independent runs to achieve more accurate comparisons.

### 4.3. Experimental Results and Analysis

**Figure 8:**
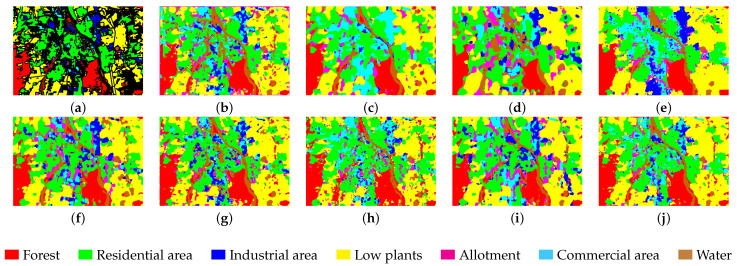
Visualization results of different model classifications in the Augsburg dataset. (**a**) Ground truth. (**b**) SPRN. (**c**) CLOLN. (**d**) FDGC. (**e**) WFCG. (**f**) SSFTT. (**g**) GSC-VIT. (**h**) MambaHSI. (**i**) IGroupSS-Mamba. (**j**) Ours. The color markers below indicate category correspondences.

**Figure 9:**
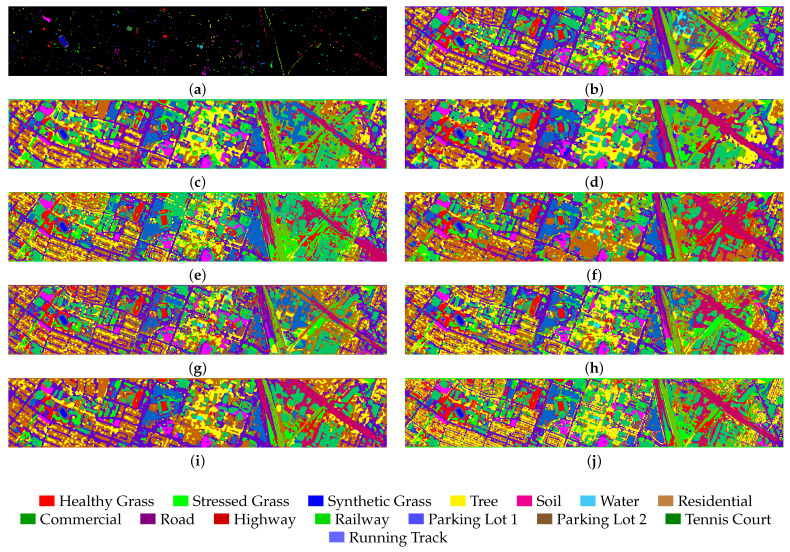
Visualization results of different model classifications in the Houston2013 dataset. (**a**) Ground truth. (**b**) SPRN. (**c**) CLOLN. (**d**) FDGC. (**e**) WFCG. (**f**) SSFTT. (**g**) GSC-VIT. (**h**) MambaHSI. (**i**) IGroupSS-Mamba. (**j**) Ours. The color markers below indicate category correspondences.

**Figure 10:**
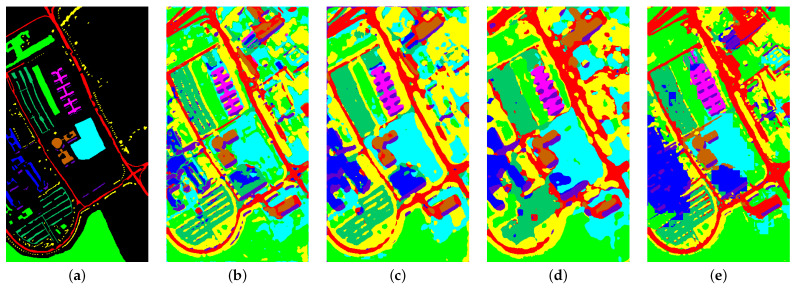

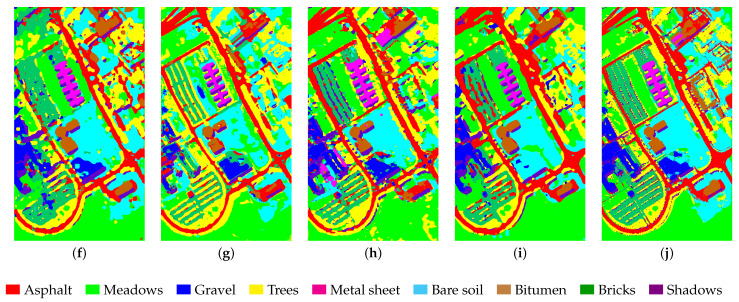
Visualization results of different model classifications in the Pavia University dataset. (**a**) Ground truth. (**b**) SPRN. (**c**) CLOLN. (**d**) FDGC. (**e**) WFCG. (**f**) SSFTT. (**g**) GSC-VIT. (**h**) MambaHSI. (**i**) IGroupSS-Mamba. (**j**) Ours. The color markers below indicate category correspondences.

**Figure 11:**
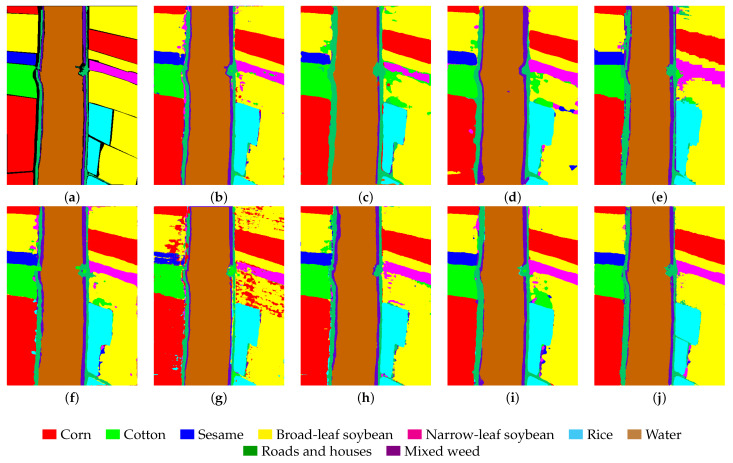
Visualization results of different model classifications in the WHU-Hi-LongKou dataset. (**a**) Ground truth. (**b**) SPRN. (**c**) CLOLN. (**d**) FDGC. (**e**) WFCG. (**f**) SSFTT. (**g**) GSC-VIT. (**h**) MambaHSI. (**i**) IGroupSS-Mamba. (**j**) Ours. The color markers below indicate category correspondences.

We selected eight models based on four different categories for comparison. These models include SPRN [36] and CLOLN [37] based on CNN, SSFTT [38] and GSC-VIT [39] based on Transformer networks, FDGC [40] and WFCG [41] based on GCN, and MambaHSI [42] and IGroupSS-Mamba [43] based on Mamba networks. Finally, all experiments were conducted under the parameter settings specified in the original paper, using the original hyperparameters to ensure the fairest and most accurate comparison results. The experimental results are the average and variance of ten runs with the specific comparison of experimental results presented in Table 5, Table 6, Table 7 and Table 8. As shown in the tables, our proposed model achieves the highest OA, AA, and Kappa across all datasets as well as the parameters (K) and FLOPs (M) for each model in the experiments. The results are visualized in Figure 8, Figure 9, Figure 10 and Figure 11. In the following subsections, we will analyze the classification performance of different models on the selected datasets.

#### 4.3.1. Results and Analysis on the Augsburg Dataset

As shown in Table 5, the proposed model achieves the highest performance on OA, AA, and Kappa with respective values of 90.14%, 83.87%, and 86.26%. Compared to the second-best model, IGroupSS-Mamba, our model outperforms it by 2.69% on OA, 1.11% on AA, and 3.53% on Kappa. Meanwhile, we can observe that the GCN-based model FDGC did not achieve good results with an OA of only 77.37%. This may be because GCN relies on iterative learning from neighboring nodes, and when the number of selected samples is small, it is difficult to fully reflect the actual graph structure, which affects feature learning and classification performance. However, the WFCG model achieved better results than the FDGC model. This may be because modifying the traditional GCN to a Graph Attention Network (GAT) enables it to adaptively learn the attention weights between nodes, effectively capturing the complex local dependencies between pixels and thereby improving classification accuracy. Additionally, the SSFTT model based on Transformers, which relies on self-attention mechanisms, can model pixel relationships across the entire domain, outperforming CNN-based models such as SPRN and CLOLN. While the CNN-based model CLOLN does not lead in accuracy, it has the lowest number of parameters and FLOPs, which can be attributed to the CNN’s local perception and weight-sharing design, resulting in significantly fewer parameters and FLOPs compared to fully connected networks. Regarding parameter count and FLOPs, the proposed model also holds a leading position with a classification accuracy superior to that of other models. From Figure 8, we can see that compared to other comparison models, our proposed model yields smoother classification results and the least noise.

#### 4.3.2. Results and Analysis on the Houston2013 Dataset

As shown in Table 6, compared to the AU dataset FDGC model, the accuracy has improved on this dataset, which also verifies that the GCN relies on the propagation characteristics of the graph structure, specifically the number of samples. Meanwhile, the Mamba-based model IGroupSS-Mamba still achieved the second-best performance, proving the Mamba module’s effectiveness compared to other models. Meanwhile, we can see that while the WFCG model achieves an intermediate level of classification accuracy, it requires a massive amount of FLOPS, reaching 92,600.4 M, reflecting the significant increase in FLOPS required by GAT as the dataset grows. CLOCN continues to maintain the lowest parameter count and FLOPS. The model we proposed still achieves the highest classification accuracy with the OA reaching 96.63%, the AA reaching 97.11%, and Kappa reaching 96.36%. As shown in Figure 9, our model still achieves optimal classification performance and remains highly accurate even when dealing with smaller target trees. However, SSFTT exhibits a higher rate of classification errors when dealing with similar targets such as Parking lot 1 and Parking lot 2.

#### 4.3.3. Results and Analysis on the Pavia University Dataset

As shown in Table 7, the proposed model maintains the best performance in terms of accuracy with OA, AA, and Kappa values all exceeding 98%. At the same time, the parameters and FLPOS of the proposed model are second only to those of the CNN-based model CLOLN, achieving the second-best performance and high accuracy while maintaining low computational complexity. We found that the classification accuracy of IGroupMamba, which has consistently ranked second in the AU and HU2013 datasets, has decreased. This may be because when the dataset contains more complex feature mixtures or increased spatial resolution differences, the difficulty of feature extraction increases, and the interval group space–spectral blocks of IGroupSS-Mamba cannot effectively capture the spatial–spectral context information, leading to a decline in model performance. Additionally, we observed that Mamba-based models outperform those based on the Transformer. This may be because Mamba models, which utilize a selective state space sequence modeling mechanism, can better capture long-range dependencies in long sequences compared to the self-attention mechanism of Transformers, thereby achieving higher accuracy. As shown in Figure 10, our proposed model exhibits distinct features at object edges, achieving the best classification performance. Meanwhile, as shown in Table 9, our proposed model achieved a training time of 9.51 s and a testing time of 0.48 s, delivering the second-best performance among all comparison models. This demonstrates that our model attains optimal results within a shorter training cycle, further validating its superiority.

#### 4.3.4. Results and Analysis on the WHU-Hi-LongKou Dataset

As shown in Table 8, even with the LK dataset, which consists of large images but a small number of training samples, our model outperforms other models. Its OA, AA, and Kappa values are as high as 98.51%, 98.60%, and 98.04%, respectively. Additionally, we can observe that our model achieves the highest classification accuracy across six categories. Furthermore, the OA accuracy of the Mamba-based model exceeds 97%, further validating the advantage of the Mamba model over other models in large-scale classification tasks. Finally, as shown in Figure 11, the GSC-VIT model exhibits a high error rate in the broadleaf soybean category with significant noise evident in the visualization. Additionally, we can observe that other models exhibit numerous classification errors along the edges in the corn category. In contrast, our proposed model yields the smoothest and most accurate results, further confirming its superiority.

## 5. Discussion

### 5.1. Impact of the Patch Size

The range of patch sizes determines the strength of the spatial context information captured. To quantitatively assess the impact of different patch sizes on the proposed model’s performance, this experiment fixed other parameters and only altered the spatial dimensions of the input patches, setting them to 9, 11, 13, 15, and 17. The changes in OA were tested on the AU, HU2013, PU, and LK datasets.

**Figure 12:**
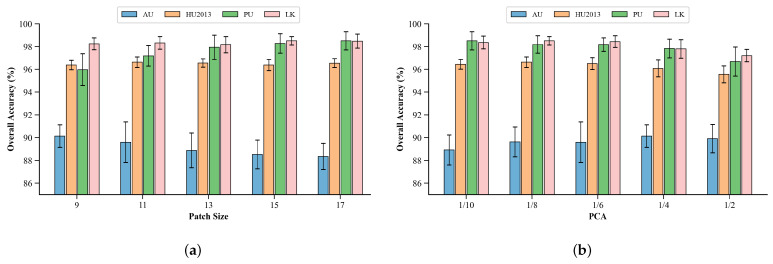
The impact of patch size and PCA on model performance. (**a**): Patch size, (**b**): PCA.

As shown in Figure 12a, the different characteristics of the datasets also dominate the differences in their classification accuracy. The PU and LK datasets achieve higher accuracy at larger patch sizes, so increasing the patch size can provide more spatial context to improve accuracy. The HU2013 dataset, on the other hand, exhibits relatively stable accuracy at medium patch sizes, while excessively large or small patch sizes tend to lead to a decline in accuracy. The AU dataset exhibits relatively stable accuracy at smaller patch sizes with accuracy gradually decreasing as the patch size increases. These results suggest that appropriate patch sizing balances spatial context with feature redundancy.

### 5.2. Impact of PCA on the Results

To quantify the impact of the PCA ratio on the performance of the proposed model, this experiment fixed the network structure and set five different PCA ratios: 1/10, 1/8, 1/6, 1/4, and 1/2. For each PCA ratio, the changes in OA were analyzed across four datasets.

As shown in Figure 12b, the PCA ratio of 1/8 is the relatively optimal ratio. At this point, PU and LK maintain peak accuracy, while HU2013 accuracy is improved to a maximum value of 96.63%. At this point, the AU error bar is the shortest, indicating its optimal stability. This ratio removes redundant noise from AU and HU2013 while retaining the key spectral features of PU and LK. This setting effectively removes redundant spectral information while retaining essential discriminative features, enhancing the efficiency of spectral representation and improving downstream classification.

### 5.3. The Impact of Embedding Dimensions

In HSIC, the embedding dimension (Embedding) determines the model’s ability to encode spectral–spatial features. To investigate its impact on performance, five groups of embedding dimensions were set: 32, 64, 96, 128, and 160. The OA, AA, and Kappa changes were analyzed across the AU, HU2013, PU, and LK datasets.

**Figure 13:**
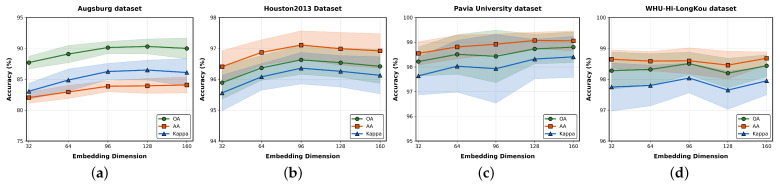
The impact of different embedding dimensions on four datasets. (**a**): Augsburg dataset, (**b**): Houston2013 dataset, (**c**): Pavia University dataset, (**d**): WHU-Hi-LongKou dataset.

As shown in Figure 13, at lower dimensions (32–64), the OA, AA, and Kappa values of all datasets typically increase. This is because at lower dimensions, the feature encoding capacity is limited, while increasing the dimension can capture more spectral–spatial correlation information, thereby improving the discriminative power of classification. Most datasets reach their performance peak at medium dimensions (64–128). This indicates that this dimension range effectively balances rich feature expression with avoiding introducing excessive redundancy (which may lead to overfitting). At higher dimensions (128–160), simpler datasets, such as PU and LK, maintain stable or slightly improved performance, as their features are easier to distinguish. However, model performance growth slows or fluctuates for complex datasets like AU and HU2013, possibly due to overly strong feature expression, which introduces noise.

### 5.4. Ablation Experiment

#### 5.4.1. The Impact of Different Modules

To validate the effectiveness of the proposed modules, we designed and conducted a series of ablation experiments. The experiments involved the application of the SGGC, SAFM, and SSAF modules. To ensure fair comparison, we simultaneously assumed that the SAFM module was replaced with a standard MLP and that the Sum module directly concatenated the spatial and spectral features in SSAF. The comparison experiments were conducted on the PU dataset, where 30 samples were used for training and the remaining samples were used as test samples. Evaluation metrics included OA, AA, and Kappa. As shown in Table 10, integrating these modules achieves the best performance, demonstrating the synergistic effect of reduced redundancy and improved spectral–spatial feature representation.

#### 5.4.2. The Impact of Different Encoder Block Numbers

To determine the effect of the number of encoder blocks on SGFNet’s performance, we varied the number of encoder blocks from 1 to 5. These experiments were conducted on the PU dataset with the number of samples in the training set fixed at 30 and the remaining samples allocated to the test set. The performance evaluation metrics were OA, AA, and Kappa, and the model parameters (K) and FLOPS (M) were also recorded.

As shown in Table 11, the experimental results exhibit a clear performance trend: as the number of encoder blocks increases, model performance gradually improves until it reaches a peak at three encoder blocks, after which further increases in the number of encoder blocks result in diminishing returns. When the number of encoders is set to three, the model achieves optimal performance across all metrics. It also has a relatively balanced computational overhead, achieving a balance between predictive capability and computational efficiency.

### 5.5. Feature Visualization

**Figure 14:**
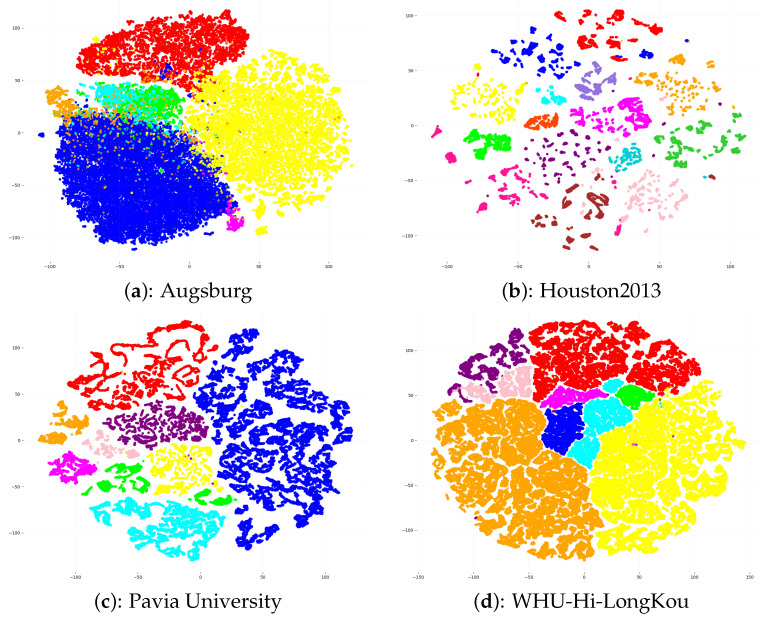
t-SNE visualisation on four different datasets.

To further demonstrate the capability of our proposed SGFNet in learning feature distributions, t-SNE [44] is employed as a dimension reduction tool to visually examine the distribution of the learned features. As shown in Figure 14, the t-SNE projection results vividly demonstrate that our model exhibits excellent feature separation capabilities across all four datasets. It can be clearly observed that features belonging to different categories are distinctly separated into mutually isolated clusters in the low-dimensional embedding space. In contrast, features within the same category remain highly compact with minimal internal variability. This indicates that the model effectively extracts discriminative spectral–spatial features while avoiding redundant or noisy representations.

## 6. Conclusions

In this paper, we propose SGFNet for HSIC, which is a network in which spectral features continuously guide the extraction of spatial features. SGFNet integrates three core components: the Spectral-Aware Filtering Module (SAFM) for enhancing key spectral information, the Spectral–Spatial Adaptive Fusion (SSAF) module for the dynamic integration of spectral and spatial features, and the Spectral Guidance Gated CNN (SGGC)—a Mamba-inspired architecture without the SSM mechanism—augmented with SSAF and spectral guidance to enable effective spatial feature extraction while maintaining a lightweight design. We investigated the feasibility of applying the MambaOut architecture to HSIC and conducted extensive experiments using four publicly available datasets. The results demonstrate that SGFNet consistently outperforms eight state-of-the-art methods across multiple evaluation metrics, highlighting the effectiveness of the proposed modules and supporting the design choice of removing the SSM mechanism. From an information–theoretic perspective, SGFNet effectively reduces redundant spectral information, which can be interpreted as lowering the entropy of the feature representations while preserving discriminative information critical for classification. This balance between redundancy reduction and information preservation contributes to the model’s superior performance and efficient representation of spectral–spatial features. In future work, we plan to extend SGFNet to other hyperspectral tasks, such as object detection and change detection, and to develop lightweight variants suitable for deployment in resource-constrained environments.

## Figures and Tables

**Table 1:** The land cover categories and the dataset division for each category of the Augsburg dataset.

Class	Category	Training	Testing	Total
1	Forest	30	13,477	13,507
2	Residential area	30	30,299	30,329
3	Industrial area	30	3821	3851
4	Low plants	30	26,827	26,857
5	Allotment	30	545	575
6	Commercial area	30	1615	1645
7	Water	30	1500	1530
Total		210	78,084	78,294

**Table 2:** The land cover categories and the dataset division for each category of the Houston2013 dataset.

Class	Category	Training	Testing	Total
1	Healthy grass	30	1221	1251
2	Stressed grass	30	1224	1254
3	Synthetic grass	30	667	697
4	Tree	30	1214	1244
5	Soil	30	1212	1242
6	Water	30	295	325
7	Residential	30	1238	1268
8	Commercial	30	1214	1244
9	Road	30	1222	1252
10	Highway	30	1197	1227
11	Railway	30	1205	1235
12	Parking lot 1	30	1203	1233
13	Parking lot 2	30	439	469
14	Tennis court	30	398	428
15	Running track	30	630	660
Total		450	14,579	15,029

**Table 3:** The land cover categories and the dataset division for each category of the Pavia University dataset.

Class	Category	Training	Testing	Total
1	Asphalt	30	6601	6631
2	Meadows	30	18,619	18,649
3	Gravel	30	2069	2099
4	Trees	30	3034	3064
5	Metal Sheets	30	1315	1345
6	Bare-soil	30	4999	5029
7	Bitumen	30	1300	1300
8	Bricks	30	3652	3682
9	Shadows	30	917	947
Total		270	42,506	42,776

**Table 4:** The land cover categories and the dataset division for each category of the WHU-Hi-LongKou dataset.

Class	Category	Training	Testing	Total
1	Corn	30	34,481	34,511
2	Cotton	30	8344	8374
3	Sesame	30	3001	3031
4	Broad-leaf soybean	30	63,182	63,212
5	Narrow-leaf soybean	30	4121	4151
6	Rice	30	11,824	11,854
7	Water	30	67,026	67,056
8	Roads and houses	30	7094	7124
9	Mixed weed	30	5199	5229
Total		270	204,272	204,542

**Table 5:** Quantitative result (ACC% ± STD%) of Augsburg dataset. Best in **bold**.

Class	CNN-Based		GCN-Based		Transformer-Based		Mamba-Based		Ours
	SPRN	CLOLN	FDGC	WFCG	SSFTT	GSC-ViT	MambaHSI	IGroupSS-Mamba	SGFNet
Forest	94.05 ± 3.42	92.06 ± 6.36	87.84 ± 3.15	93.67 ± 2.93	95.56 ± 1.89	93.32 ± 3.73	95.51 ± 1.43	93.88 ± 2.89	**97.57 ± 1.38**
Residential area	86.47 ± 3.33	**91.80 ± 7.22**	73.77 ± 7.64	86.08 ± 3.10	86.39 ± 5.81	83.05 ± 6.35	84.02 ± 5.69	84.18 ± 3.50	87.57 ± 3.48
Industrial area	65.49 ± 5.43	**71.61 ± 16.32**	66.59 ± 7.68	70.22 ± 7.42	68.49 ± 4.04	62.22 ± 14.32	62.69 ± 8.93	69.99 ± 7.44	69.34 ± 7.13
Low plants	86.16 ± 4.05	**96.92 ± 2.17**	78.58 ± 6.55	88.80 ± 3.75	88.42 ± 5.31	86.37 ± 4.59	79.2 ± 5.77	92.04 ± 2.48	94.39 ± 2.30
Allotment	93.10 ± 3.10	15.07 ± 7.58	84.42 ± 6.01	**97.27 ± 1.77**	91.03 ± 3.13	92.36 ± 4.67	89.52 ± 4.06	94.70 ± 2.42	96.50 ± 1.74
Commercial area	66.71 ± 4.99	33.64 ± 12.04	**69.58 ± 5.89**	67.95 ± 7.07	63.42 ± 11.28	58.91 ± 13.82	64.76 ± 6.53	66.25 ± 7.59	66.69 ± 5.73
Water	73.44 ± 3.80	33.87 ± 10.07	68.44 ± 4.34	80.21 ± 2.60	68.97 ± 5.65	72.88 ± 5.01	71.53 ± 3.49	**78.28 ± 2.98**	75.03 ± 4.41
OA (%)	86.03 ± 2.29	81.24 ± 6.09	77.37 ± 4.12	87.14 ± 1.96	87.02 ± 1.00	84.31 ± 3.21	82.7 ± 2.71	87.45 ± 0.96	**90.14 ± 0.99**
AA (%)	80.77 ± 1.70	62.14 ± 4.14	75.55 ± 2.15	83.46 ± 1.09	80.33 ± 1.86	78.44 ± 1.43	78.18 ± 1.09	82.76 ± 1.23	**83.87 ± 0.89**
Kappa (%)	80.80 ± 2.97	74.52 ± 6.96	69.89 ± 4.86	82.17 ± 2.63	82.00 ± 1.24	78.52 ± 4.05	68.33 ± 8.98	82.73 ± 1.22	**86.26 ± 1.32**
Params (K)	181.37	**5.3**	1855.61	75.83	148.42	97.48	405.51	139.49	108.75
FLOPs (M)	11.75	**1.57**	27.98	23761.22	22.8	6.23	8,160.15	10.34	3.72

**Table 6:** Quantitative result (ACC% ± STD%) of Houston2013 dataset. Best in **bold**.

Class	CNN-Based		GCN-Based		Transformer-Based		Mamba-Based		Ours
	SPRN	CLOLN	FDGC	WFCG	SSFTT	GSC-ViT	MambaHSI	IGroupSS-Mamba	SGFNet
Healthy Grass	97.26 ± 3.93	88.24 ± 9.00	91.48 ± 3.66	93.85 ± 5.44	90.74 ± 6.77	88.32 ± 19.51	95.20 ± 4.66	**99.05 ± 0.66**	96.21 ± 2.73
Stressed Grass	96.16 ± 3.63	93.41 ± 5.99	92.03 ± 3.52	96.74 ± 4.29	91.96 ± 13.40	93.50 ± 5.28	98.29 ± 1.26	**99.16 ± 0.90**	98.60 ± 1.33
Synthetic Grass	99.27 ± 0.63	95.21 ± 9.84	99.27 ± 0.60	99.93 ± 0.10	99.50 ± 0.67	99.16 ± 1.32	99.74 ± 0.46	99.73 ± 0.06	**99.99 ± 0.04**
Tree	95.07 ± 2.65	97.44 ± 3.80	90.84 ± 5.25	96.46 ± 2.34	91.50 ± 5.64	93.50 ± 2.15	97.26 ± 2.47	98.57 ± 1.11	**99.28 ± 1.71**
Soil	99.98 ± 0.05	97.07 ± 2.86	99.64 ± 0.58	99.93 ± 0.14	99.59 ± 0.66	98.81 ± 1.93	99.54 ± 0.76	99.99 ± 0.02	**100.00 ± 0.00**
Water	98.75 ± 1.07	94.85 ± 4.72	96.36 ± 4.37	96.61 ± 4.65	95.48 ± 6.23	97.56 ± 3.38	97.47 ± 2.33	**100.00 ± 0.00**	98.71 ± 3.86
Residential	93.23 ± 2.58	94.02 ± 2.94	84.64 ± 5.10	93.63 ± 6.75	84.99 ± 7.97	87.50 ± 6.28	93.03 ± 2.25	94.26 ± 3.86	**96.03 ± 2.79**
Commercial	82.63 ± 4.75	**93.80 ± 3.94**	83.25 ± 4.96	87.93 ± 4.27	82.21 ± 7.55	78.39 ± 6.59	81.35 ± 3.98	88.54 ± 1.75	88.50 ± 4.75
Road	88.96 ± 3.32	88.32 ± 2.83	88.02 ± 3.93	87.72 ± 6.49	84.31 ± 4.78	81.96 ± 3.99	90.03 ± 3.40	84.04 ± 7.21	**91.42 ± 3.90**
Highway	94.82 ± 4.24	76.55 ± 6.96	96.35 ± 1.70	90.44 ± 10.33	92.79 ± 6.77	92.64 ± 7.93	96.55 ± 1.49	92.13 ± 3.15	**97.51 ± 2.66**
Railway	91.61 ± 2.86	91.57 ± 4.40	95.86 ± 2.82	93.13 ± 5.63	92.69 ± 9.60	79.84 ± 8.92	92.74 ± 2.18	96.37 ± 1.86	**98.31 ± 2.52**
Parking Lot 1	91.28 ± 4.23	83.37 ± 6.17	88.97 ± 4.39	92.31 ± 3.08	84.53 ± 12.86	84.66 ± 9.62	91.19 ± 3.68	**96.55 ± 1.51**	95.15 ± 2.85
Parking Lot 2	92.23 ± 4.09	89.11 ± 4.64	95.09 ± 3.64	93.99 ± 3.92	89.82 ± 11.24	94.72 ± 2.82	**97.67 ± 2.04**	97.60 ± 1.38	97.02 ± 2.23
Tennis Court	**100.00 ± 0.00**	97.98 ± 2.63	99.66 ± 0.56	**100.00 ± 0.00**	**100.00 ± 0.00**	99.87 ± 0.30	**100 ± 0.00**	**100.00 ± 0.00**	**100.00 ± 0.00**
Running Track	99.73 ± 0.55	96.08 ± 3.96	98.70 ± 1.78	100.00 ± 0.00	99.86 ± 0.14	99.78 ± 0.32	**100 ± 0.00**	**100.00 ± 0.00**	**100.00 ± 0.00**
OA (%)	93.95 ± 0.79	90.31 ± 1.73	92.34 ± 0.83	94.02 ± 1.50	91.39 ± 3.23	89.67 ± 2.71	94.46 ± 0.83	95.64 ± 0.72	**96.63 ± 0.47**
AA (%)	94.73 ± 0.66	91.80 ± 1.46	93.45 ± 0.96	94.83 ± 1.49	92.55 ± 3.14	91.35 ± 2.20	95.34 ± 0.78	96.40 ± 0.61	**97.11 ± 0.46**
Kappa (%)	93.45 ± 0.85	89.52 ± 1.87	91.61 ± 1.02	93.59 ± 1.64	90.62 ± 3.56	88.83 ± 2.92	94.21 ± 1.93	95.28 ± 0.78	**96.36 ± 0.51**
Params (K)	182.35	**4.85**	2383.68	71.67	148.42	88.78	401.94	340.07	105.21
FLOPs (M)	11.71	**1.37**	29.46	92,600.4	22.8	5.64	27,681.67	31.41	5.34

**Table 7:** Quantitative result (ACC% ± STD%) of Pavia University dataset. Best in **bold**.

Class	CNN-Based		GCN-Based		Transformer-Based		Mamba-Based		Ours
	SPRN	CLOLN	FDGC	WFCG	SSFTT	GSC-ViT	MambaHSI	IGroupSS-Mamba	SGFNet
Asphalt	92.82 ± 3.00	98.27 ± 1.00	88.35 ± 6.60	**98.68 ± 1.05**	88.01 ± 7.66	97.10 ± 1.64	94.35 ± 1.73	96.98 ± 1.27	97.21 ± 2.88
Meadows	85.90 ± 6.04	**98.98 ± 0.76**	94.52 ± 2.92	96.28 ± 2.02	96.34 ± 3.12	97.68 ± 1.32	96.43 ± 1.92	93.11 ± 1.60	98.56 ± 1.14
Gravel	91.11 ± 6.57	87.83 ± 7.54	88.81 ± 2.95	**99.70 ± 0.63**	89.44 ± 9.31	86.46 ± 7.42	93.39 ± 5.35	95.00 ± 1.77	97.93 ± 1.79
Trees	94.94 ± 1.68	97.80 ± 1.46	85.60 ± 4.60	94.85 ± 3.32	92.79 ± 4.86	91.83 ± 9.94	89.75 ± 3.60	96.48 ± 0.48	**98.73 ± 0.89**
Metal sheets	99.76 ± 0.26	99.68 ± 0.59	97.28 ± 3.16	**100.00 ± 0.00**	99.79 ± 0.22	99.45 ± 0.53	99.99 ± 0.02	99.96 ± 0.06	99.93 ± 0.13
Bare soil	93.79 ± 4.59	85.27 ± 7.50	97.85 ± 2.17	99.01 ± 0.83	97.14 ± 3.34	79.65 ± 9.24	98.60 ± 0.93	**99.27 ± 1.28**	99.00 ± 0.86
Bitumen	97.65 ± 3.46	85.92 ± 7.00	95.54 ± 10.96	99.82 ± 0.27	98.81 ± 2.30	86.63 ± 10.10	96.50 ± 3.13	99.85 ± 0.19	**99.96 ± 0.05**
Bricks	91.45 ± 2.81	90.93 ± 4.12	91.42 ± 5.07	98.59 ± 1.11	89.11 ± 5.48	89.50 ± 4.70	94.40 ± 2.93	92.93 ± 4.89	**98.89 ± 0.61**
Shadows	99.33 ± 0.61	97.12 ± 2.03	88.33 ± 5.31	99.01 ± 1.57	98.24 ± 0.77	98.71 ± 1.10	**99.36 ± 0.62**	98.65 ± 0.61	99.05 ± 0.54
OA (%)	90.36 ± 3.02	94.97 ± 1.43	92.76 ± 2.26	97.52 ± 1.03	94.11 ± 1.59	92.65 ± 2.30	95.74 ± 0.90	95.29 ± 0.81	**98.51 ± 0.80**
AA (%)	94.08 ± 1.31	93.53 ± 1.59	91.97 ± 3.00	98.44 ± 0.54	94.22 ± 2.00	91.89 ± 1.73	95.86 ± 1.11	96.92 ± 0.40	**98.81 ± 0.48**
Kappa (%)	87.53 ± 3.81	93.38 ± 1.84	90.49 ± 2.93	96.74 ± 1.34	92.14 ± 2.33	90.38 ± 2.95	95.00 ± 2.24	93.84 ± 1.04	**98.03 ± 1.05**
Params (K)	179.02	**4.1**	1987.61	65.95	148.42	77.9	412.24	139.55	48.47
FLOPs (M)	11.55	**1.15**	28.34	26,502.92	22.8	4.96	25,746.48	10.34	5.74

**Table 8:** Quantitative result (ACC% ± STD%) of WHU-Hi-LongKou dataset. Best in **bold**.

Class	CNN-Based		GCN-Based		Transformer-Based		Mamba-Based		Ours
	SPRN	CLOLN	FDGC	WFCG	SSFTT	GSC-ViT	MambaHSI	IGroupSS-Mamba	SGFNet
Corn	98.90 ± 1.69	96.38 ± 4.65	97.14 ± 2.11	99.28 ± 0.53	99.21 ± 0.57	97.2 ± 2.85	**99.32 ± 0.54**	99.21 ± 0.40	99.31 ± 0.50
Cotton	97.96 ± 1.86	93.69 ± 13.25	95.70 ± 2.59	97.26 ± 0.91	98.38 ± 1.37	95.73 ± 2.67	97.65 ± 2.2	98.21 ± 0.77	**98.78 ± 0.90**
Sesame	99.28 ± 0.49	70.09 ± 31.51	98.74 ± 0.90	99.08 ± 0.51	99.39 ± 0.83	91.94 ± 14.93	99.28 ± 1.13	99.41 ± 0.41	**99.41 ± 0.53**
Broad-leaf soybean	95.75 ± 1.41	**99.57 ± 0.28**	92.34 ± 2.53	93.74 ± 1.41	95.85 ± 2.22	87.64 ± 10.14	93.52 ± 2.01	95.84 ± 1.35	96.58 ± 1.13
Narrow-leaf soybean	95.39 ± 4.56	68.34 ± 22.10	98.57 ± 1.32	99.36 ± 0.84	98.57 ± 1.32	97.74 ± 1.67	98.07 ± 2.3	98.81 ± 0.47	**99.67 ± 0.35**
Rice	99.04 ± 0.82	97.66 ± 1.53	94.84 ± 4.75	97.36 ± 2.78	97.82 ± 1.89	98.00 ± 3.34	98.75 ± 0.7	**99.38 ± 0.27**	99.25 ± 0.36
Water	99.10 ± 0.79	99.47 ± 0.46	96.07 ± 1.66	98.79 ± 0.69	98.18 ± 1.01	99.52 ± 0.22	99.73 ± 0.17	99.02 ± 0.68	**99.84 ± 0.15**
Roads and houses	94.46 ± 2.56	77.41 ± 15.33	86.86 ± 4.32	97.18 ± 2.22	92.61 ± 5.81	93.70 ± 2.84	91.61 ± 3.34	96.27 ± 0.61	**97.66 ± 1.39**
Mixed weed	92.69 ± 3.06	73.30 ± 18.70	89.57 ± 7.44	96.55 ± 3.02	91.52 ± 8.22	93.25 ± 3.78	94.32 ± 4.61	94.12 ± 4.82	**96.93 ± 3.84**
OA (%)	97.59 ± 0.49	93.78 ± 1.94	94.62 ± 0.98	97.07 ± 0.64	97.28 ± 0.59	94.71 ± 3.06	97.14 ± 0.61	97.84 ± 0.50	**98.51 ± 0.37**
AA (%)	96.95 ± 0.57	86.22 ± 3.66	94.43 ± 1.43	97.62 ± 0.45	96.61 ± 0.72	94.97 ± 1.55	96.92 ± 0.53	97.81 ± 0.52	**98.60 ± 0.42**
Kappa (%)	96.84 ± 0.63	91.96 ± 2.41	93.02 ± 1.26	96.18 ± 0.82	96.45 ± 1.07	93.19 ± 3.8	96.25 ± 11.25	97.18 ± 0.64	**98.04 ± 0.48**
Params (K)	184.89	**6.77**	1987.61	87.66	148.42	173.06	433.62	139.55	112.11
FLOPs (M)	11.96	**2.06**	28.34	37,681.55	22.8	10.25	32,012.85	10.34	9.54

**Table 9:** Comparison of runtime on the Pavia University dataset. $T_{tr}\left(s\right)$ indicates training time, while $T_{te}\left(s\right)$ indicates the time required to test the entire HSI.

Metrics	SPRN	CLOLN	FDGC	WFCG	SSFTT	GSC-ViT	MambaHSI	IGroupSS-Mamba	Ours
$T_{tr}\left(s\right)$	9.74	9.81	5.65	328.1	11.26	13.66	384.12	52.41	9.51
$T_{te}\left(s\right)$	7.06	1.82	7.49	1.71	3.25	4.89	0.04	27.27	0.48

**Table 10:** Ablation experiment results. **Bold** indicates the best results; the symbols in the first row denote module names; ✓ and × indicate whether the module is included.

SGGC	MLP	SAFM	Sum	SSAF	OA (%)	AA (%)	Kappa (%)
✓	×	×	×	×	97.01 ± 1.48	97.26 ± 1.02	96.05 ± 1.93
✓	✓	×	✓	×	97.59 ± 0.74	98.08 ± 0.54	96.82 ± 0.97
✓	×	✓	✓	×	97.74 ± 0.73	98.19 ± 0.75	97.02 ± 0.95
✓	✓	×	×	✓	98.15 ± 0.53	98.37 ± 0.73	97.55 ± 0.70
✓	×	✓	×	✓	**98.51 ± 0.80**	**98.81 ± 0.48**	**98.03 ± 1.05**

**Table 11:** Model performance under different encoder block numbers.

Number	OA (%)	AA (%)	Kappa (%)	Params (K)	FLOPS (M)
1	97.28 ± 0.88	97.77 ± 0.78	96.41 ± 1.16	20.73	3.64
2	98.23 ± 0.66	98.44 ± 0.76	97.65 ± 0.88	34.6	4.52
3	98.51 ± 0.80	98.81 ± 0.48	98.03 ± 1.05	48.47	5.74
4	98.36 ± 0.53	98.70 ± 0.32	97.82 ± 0.70	62.34	6.64
5	97.94 ± 1.06	98.41 ± 0.53	97.28 ± 1.39	76.22	8.06

## Data Availability

The original contributions presented in this study are included in the article. Further inquiries can be directed to the corresponding author(s).
